# Advances in personalized radiotherapy

**DOI:** 10.1186/s12885-024-12317-3

**Published:** 2024-05-03

**Authors:** Venkata SK. Manem, Farzad Taghizadeh-Hesary

**Affiliations:** 1 Centre de Recherche du CHU de Québec - Université Laval, Quebec, Canada; 2 Department of Mathematics and Computer Science, University of Quebec at Trois-Rivières, Quebec, Canada; 3 ENT and Head and Neck Research Center and Department, The Five Senses Health Institute, School of Medicine, Iran University of Medical Sciences, Tehran, Iran; 4 Department of Radiation Oncology, Iran University of Medical Sciences, Tehran, Iran

## Abstract

Radiotherapy is a mainstay of cancer treatment. The clinical response to radiotherapy is heterogeneous, from a complete response to early progression. Recent studies have explored the importance of patient characteristics in response to radiotherapy. In this editorial, we invite contributions for a BMC Cancer collection of articles titled ‘Advances in personalized radiotherapy’ towards the improvement of treatment response.

## Main text

Cancer is a heterogeneous disease in the sense of different genomic, epigenomic, transcriptomic, and proteomic characteristics of the cancer cells. Furthermore, host characteristics, such as age, gender, and even biological and lifestyle characteristics, may impact treatment response and toxicities. Therefore, treatment options should be individualized based on the tumor and host characteristics to avoid overtreatment and undertreatment.

“Knowing the sort of *person* who has a disease is more crucial than knowing the sort of *disease* a person has” Hippocrates 440 − 360 BC.

This ancient quote has been revived in nowadays clinical practice. Generally, the clinical outcomes of cancer treatments constitute a wide range, from a complete response to early progression. This issue has resulted in a global march from the *one-size-fits-all* approach towards *risk-adapted* therapies [1]. In other words, physicians must know the prognosis and risk of disease recurrence to tailor treatments to individual patients. This paradigm has evolved into an approach in oncology called *personalized oncology*. According to the American Cancer Society, personalized oncology (a.k.a. precision oncology) is a way that healthcare providers can offer and plan specific cancer care for their patients based on specific gene mutations and proteins. Thanks to the recent advances in molecular biology, this approach has been increasingly employed to treat different malignancies.

Genetic instabilities in proto-oncogenes and tumor suppressor genes are among the hallmarks of cancer. Therefore, malignant tumors can express different proteins in different patients. This inherent variability of cancer has led to the development of personalized treatment [2]. In this approach, treatment is de-escalated in some cases to improve patients’ quality of life and reduce treatment toxicities, health system burdens, and financial costs. On the other hand, escalated therapies can be selected in high-risk patients to reduce disease recurrence. The following example regarding breast cancer denotes the evolution of cancer treatments during the last decades: The trend from post-lumpectomy, full-dose, conventionally-fractionated, whole-breast irradiation in all patients (in the 1980s) to no adjuvant radiotherapy or employing partial breast, hypofractionated regimens in the low-risk groups (in 2020s) [3]; from adjuvant chemotherapy in all patients with larger than one-centimeter breast cancer (2013) to no adjuvant chemotherapy in selected patients with lymphatic involvement (2022) [4]; and from axillary lymph node dissection for all breast cancer patients (in 1509, introduced by Michael Servetus) to waiving sentinel lymph node biopsy in elderly patients with early-stage disease (2013) [5]. In these (and similar) cases, personalized treatments have benefited the patients with fewer treatment toxicities while maintaining efficacy.

For more than half of patients with cancer, radiotherapy is an integral part of their treatment plan. Radiotherapy is based on the concept of delivering the maximum dose to the target volume while sparing the normal tissues. The available radiotherapy guidelines are mainly based on the population averages. This approach faces the physician with a two-fold issue: (a) tumors are heterogeneous with different genetic and epigenetic factors, and (b) individuals harboring tumors differ in demographic, racial, lifestyle, and genetic factors [6]. Therefore, radiotherapy must be tailored according to the tumor’s and host’s factors.

The pre-requisite of risk-adapted therapies is to find risk-determining factors, also called *prognostic factors*. According to the National Cancer Institute, prognostic factors are conditions or patient characteristics applied to estimate the chance of recovery or recurrence. Besides, physicians need to determine the *predictive factors* to predict the response to a specific treatment. To this end, different disciplines have joined this movement to find the determining factors. For example, research efforts have been underway to develop data-driven models to predict tumor radiosensitivity [7–10], along with designing different nanoparticles to improve tumor radiosensitivity [11]. A deep dive into cancer biology has expanded the multifaceted variables by introducing microscopic factors, such as tumor microenvironment components [12], cancer cells’ mitochondria [13], and different biological markers [14]. Interestingly, recent studies have delineated the importance of the gut microbiome in response to radiotherapy [15].

Despite great achievements in the last decades, personalized radiotherapy is still in its infancy, and further studies are warranted for clinical translation. This Collection calls for novel original studies in the field of personalized radiotherapy. We hope this Collection will provide a useful platform to share recent advances in radiosensitivity for all healthcare providers working in the field of oncology to improve the prognosis of patients with cancer.

## Data Availability

Not applicable.
